# Reducing hospital acquired pressure injury in a learning health center: Making the case for quality

**DOI:** 10.1002/lrh2.10355

**Published:** 2022-12-15

**Authors:** Shea Polancich, Patricia Patrician, Rebecca Miltner, Katherine Meese, Amy Armstrong, Shannon Layton, Ross Vander Noot, Terri Poe, Allyson G. Hall

**Affiliations:** ^1^ University of Alabama at Birmingham School of Nursing Birmingham Alabama USA; ^2^ University of Alabama at Birmingham Hospital Birmingham Alabama USA; ^3^ School of Health Professions University of Alabama at Birmingham Hospital Birmingham Alabama USA; ^4^ Heersink School of Medicine University of Alabama at Birmingham Hospital Birmingham Alabama USA

**Keywords:** continuous improvement, hospital acquired pressure injury (HAPI), learning health system, quality improvement, value equation

## Abstract

**Introduction:**

The purpose of this descriptive study is to examine a learning health system (LHS) continuous improvement and learning approach as a case for increased quality, standardized processes, redesigned workflows, and better resource utilization. Hospital acquired pressure injuries (HAPI) commonly occur in the hospitalized patient and are costly and preventable. This study examines the effect of a LHS approach to reducing HAPI within a large academic medical center.

**Methods:**

Our learning health center implemented a 6‐year series of iterative improvements that included both process and technology changes, with robust data and analytical reforms. In this descriptive, observational study, we retrospectively examined longitudinal data from April 1, 2018 to March 31, 2022, examining the variables of total number of all‐stage HAPI counts and average length of stay (ALOS). We also analyzed patient characteristics observed/expected mortality ratios, as well as total patient days, and the case‐mix index to determine whether these factors varied over the study period. We used the Agency for Healthcare Research and Quality cost estimates to identify the estimated financial benefit of HAPI reductions on an annualized basis.

**Results:**

HAPI per 1000 patient days for FY 20 (October 1‐September 30) and FY 21, decreased from 2.30 to 1.30 and annualized event AHRQ cost estimates for HAPI decreased by $4 786 980 from FY 20 to FY 21. A strong, statistically significant, negative and seemingly counterintuitive correlation was found (*r* = −.524, *P* = .003) between HAPI and ALOS.

**Conclusions:**

The LHS efforts directed toward HAPI reduction led to sustained improvements during the study period. These results demonstrate the benefits of a holistic approach to quality improvement offered by the LHS model. The LHS model goes beyond a problem‐based approach to process improvement. Rather than targeting a specific problem to solve, the LHS system creates structures that yield process improvement benefits over a continued time period.

## 1 INTRODUCTION

The Learning Health System (LHS) is characterized as an organization that integrates internal and external data and evidence to change processes and practices, leading to higher quality, and safer patient care.^1^ LHSs have additional characteristics that include leadership commitment to continuous improvement and a culture of learning, a systematic approach to gathering data and evidence, and use of technology to improve decision support, where data are used to refine processes and create a feedback loop for continuous improvement.^2^, ^3^ Another important characteristic of LHSs is their patient‐centeredness, or the inclusion of patients and families as members of the care team.^4^ The results of such an approach are designed to achieve higher quality, safe, and more efficient organizations that attract and retain patients and talented health care workers.^2^

**FIGURE 1:**
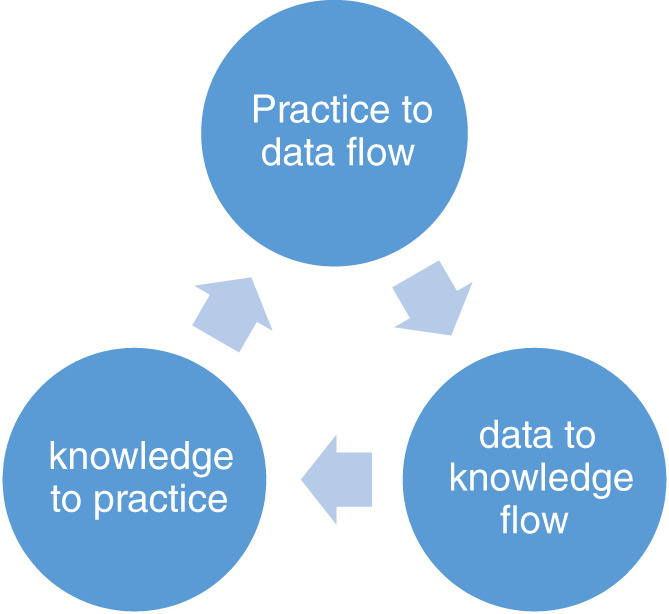
LHS three‐phase approach

Within an LHS, leaders respond to problems or focus areas systematically in three phases: (1) Practice to Data Flow, (2) Data to Knowledge Flow, and (3) Knowledge to Practice Flow^5^ Figure 1.

These phases represent a thoughtful and data‐driven approach to improvement or the development of solutions to an opportunity or challenge within a health care system and incorporate eight steps within the phases: (1) Collect Data, (2) Assemble Data, (3) Analyze Data, (4) Interpret Results, (5) Represent Knowledge, (6) Manage Knowledge, (7) Apply Knowledge, and (8) Take Action to Change Practice. Practice‐based opportunities are the focus of LHSs with actionable data from the point of care providing the impetus for continuous improvement, innovation, and organizational learning.

In a LHS, health care quality is dependent on a myriad of factors that can be categorized according to Donabedian's model of structure, process, and outcomes.^6^ The organizational structure within which care delivery occurs is composed of the physical environment, people, policies, and culture, and physical resources, such as equipment and supplies, all contributing to the quality of care. Processes or workflows are the engine for care delivery, the mechanics of quality, while the outcomes are the clinical, financial, and operational effects of quality.

Since the seminal publication “Crossing the Quality Chasm,” health care leaders were encouraged to commit to the pursuit of high quality and excellent patient outcomes.^7^ Although the health care industry's value proposition is often calculated equally by quality and cost, when there is a lapse in quality, costs increase.^8^ Likewise, when there is an excessive focus on cost‐cutting, quality generally suffers. Despite the positive implications for quality care delivery, there is a cost associated with high quality in health systems. Resources and human effort must be expended to attain excellence in quality care.^9^, ^10^, ^11^

From a patient perspective, quality health care is most prominently defined through clinical outcomes and the absence of adverse events. While costs are important, the patient's return to a higher or better state of health determines a safe, quality experience. There are specific and nationally recognized outcome measures within the health care industry for patient safety, including but not limited to rates associated with the development of hospital acquired pressure injuries (HAPI), patient falls, and infections associated with care. Patient safety and quality are inter‐related, and while they remain a focus for health care providers and leaders, there are indications that in the policy arena patient safety in the United States may be waning and that decline is beginning to be felt in health care settings.^12^, ^13^

In developing this study, we posed a central, critical question. Does the LHS approach create a strong case for the value of quality and safety in a health care organization? To address this question, we specifically evaluated if one acute care facility's HAPI improvements were sustained between October 1, 2019 to March 31, 2022, during the pandemic (Centers for Disease Control and Prevention, 2021).^14^

The purpose of this descriptive study is to examine one organization's continuous improvement and learning approach as a case for increased quality, standardized processes, redesigned workflows, and better resource utilization. We will describe the relationships that exist between structural and organizational characteristics that have an impact on outcomes specific to one adverse event, HAPI, within one organization.

### 1.1 Background application of the LHS approach: hospital acquired pressure injury (HAPI)

HAPI commonly occurs in the hospitalized patient, and may range from a small wound that heals fairly quickly to one that leads to sepsis and death. A pressure injury is defined as “localized damage to the skin and/or underlying soft tissue, typically occurring over a bony prominence or related to a medical or other device. Researchers at the AHRQ estimate that 2.5 million patients annually are impacted by pressure injury, with 3.5% to 4.5% of pressure injuries occurring in hospitalized patients, the majority of which may be preventable.^15^ Pressure injuries are costly; in the United States health care industry, patient expenditures for pressure injuries range from $9.1 to $11.6 billion dollars annually.^16^ Effective evidence informed clinical care by providers may mitigate the incidence of pressure injuries, thus funding agencies consider HAPI a never‐event hospital acquired condition (HAC).^17^, ^18^, ^19^ Our LHS problem of interest and focus area was improving the safety and quality of our patient care by reducing the incidence of HAPI.

Our organization has committed significant resources to HAPI reduction. We began our continuous improvement and organizational learning journey in 2016 with a small improvement research pilot to understand our internal status of the HAPI challenge, specifically staff competencies and HAPI stage attribution.^20^, ^21^ Using the 3‐phased LHS approach described earlier (Figure 1), we collected, analyzed, and interpreted data to discover that pressure injury staging was inaccurate, in some cases by as much as 50%, and our pressure injury reporting structures and systems were flawed. From this effort we had two significant learnings: first, developing staff competencies in practice is important, but we must utilize the expertise of those with the most competence in making decisions, such as the Wound, Ostomy, Continence (WOC) nurse for staging pressure injuries. Second, improvement cannot occur without actionable and accurate data collected from the point of care. These learnings directly focused our team on a series of iterative improvements, including the development of an analytical platform and resource reforms.

We used data from the initial pilot to represent, manage, and apply knowledge to implement a series of interventions that dramatically changed practices in our hospital. In 2017, the organization resourced a 16‐member WOC Team (WOCT) with 12 WOC nurses, a dedicated nurse practitioner, a physician medical director, and two wound care technicians. With this fully staffed WOCT, our WOC nurse‐to‐bed ratio decreased from 1:300 to 1:100, resulting in more comprehensive patient care and focused staff support for proactive mitigation of skin‐related injuries. In addition, the WOCT supported all‐stage attribution of pressure injuries, placing the assessment of HAPI at the level of the expert, with a resultant higher degree of accuracy in pressure injury staging data. We gained significant learnings from this iterative improvement effort. First, resources are necessary for improvement, and while adding human resources may increase cost initially, the long term benefits will largely pay for the investment. Second, we found that the investment in professional development and front‐line staff competency for clinical care is a worthy endeavor that is developed over time with expert mentoring and continuous coaching, but also creates a mechanism for additional learning and improvement.

The LHS model naturally aligns with continuous improvement. We used the phased approach iteratively to initiate continuous HAPI improvement that has been sustained for over 6 years (Table 1). The data inaccuracies identified during the initial pilot led to the development of a HAPI analytical system^21^ that transformed pressure injury reporting in our medical center, leading to actionable data available at the front line of care delivery. Our team integrated a business intelligence tool into our processes and thus converted real‐time, expert attributed HAPI staging from the point of care into transformational improvement data. From these data, we learned that we had specific opportunities to improve care delivery for higher risk and vulnerable patients. For example, tracheostomy patients were found to be at higher risk for HAPI associated with trach plates, a process that was improved using standardized protocols with specific treatment plans.

**TABLE 1:** LHS approach to HAPI reduction

LHS stage	Data to knowledge flow	Knowledge to practice	Practice to data flow
Organizational improvement intervention	**2016** Clinical Nurse Leader (CNL) pilot implemented^20^ Data collection on 2 nursing units (1 Intensive Care Unit, 1 Medical Surgical Unit) hospital acquired pressure injury (HAPI) staging information collected comparing bedside nursing staging attribution to CNL expert staging attribution **2018‐2019** HAPI analytic tool reports implementation shows variation and gaps in HAPI by unit and anatomic location **2020** RCA identification of higher risk of upper body HAPI with COVID proning, implementation of upper body silicone adhesive dressing	**2017** Wound, Ostomy, and Continence Team (WOCT) expansion to improve HAPI staging, pressure injury prevention, and provider education Interprofessional Skin Team (IST) initiated to design, implement, and communicate skin‐related improvements and interventions across disciplines more effectively **2018** HAPI analytical system completed and validated daily report developed for all stage HAPI counts^21^ **2020** Root Cause Analysis (RCA) HAPI review process developed in Center for Nursing Excellence **2021** Internal research shows reduction in COVID HAPI following institution of upper body silicone adhesive foam dressing in prone patients	**2020** Research on cost of quality for HAPI (Polancich et al., 2020)^22^ **2021** COVID research implemented to examine association between COVID and HAPI COVID research implemented to examine potential health disparities between COVID and HAPI^23^
Findings and outcomes	**2016** HAPI staging data from June–July 2016 on the pilot showed 21%‐50% incorrectly increased HAPI staging by staff nurses Need for more accurately staged and reported HAPI data Reduction in variation of HAPI staging by varying staff Staging expertise for HAPI to be examined **2018‐2019** HAPI most significantly impacting heels and sacrum Intensive care units largest proportion of HAPI Tracheostomy patients at higher risk (Montgomery et al., 2022) **2021** Internal research shows reduction in COVID HAPI following institution of upper body silicone adhesive foam dressing in proned patients **2022** 80‐90% reduction in all‐stage HAPI from baseline April 2018 to June 2022	**2017** Wound, Ostomy, and Continence (WOC) nurse‐to‐bed ratio decreased from 1:300 to 1:100 IST develops working group to improve data and reporting Informatics tool developed creating real‐time access to staged HAPI HAPI analytic tool development initiated **2018** WOC nurse staging of all stages of HAPI, a change from prior consult at stage 3 or above **2019** Reports developed within the HAPI analytic tool for staging, nursing unit, anatomic location **2020** Better identification of process challenges associated with HAPI developed on units	**2020** Organization cost associated with HAPI inconsistent and lower than literature estimates **2021** Real‐time adjustments in care result in continuous improvement during COVID Organizational disparities not evident by race, challenge associated with gender for increased risk of HAPI

Our clinical leaders began making real‐time adjustments for management and prevention of pressure injuries at the earliest stages of skin injury after determining heel and sacral pressure injury were significant risks for our patients, particularly those patients in intensive care units. Integrating preventative processes and measures, such as rounding, heel floating, observational scanning for devices, and equipment, with conjunctive use of silicone adhesive foam dressings mitigated risks. Our iterative learnings prompted our team to institute a significant feedback process using root cause analysis techniques to collect data on all of our HAPI occurrences, thus providing an in‐depth analysis of patterns in HAPI outcomes that could be mitigated and processes that were amenable to improvement.

During the COVID‐19 pandemic, our sustained improvement efforts focused on the impact of the coronavirus on HAPI. We used our HAPI data to adjust patient care delivery processes^24^ to reduce pressure injuries on COVID patients in our medical intensive care units and to evaluate opportunities for health disparities in our patient populations during the pandemic.^25^ We used our learnings from the root cause analysis process to quickly identify that patient therapeutics, such as proning were increasing the risk for upper body pressure injuries, particularly on the face and head. Clinical teams were able to mitigate this risk with silicone adhesive foam dressings that we had learned reduced HAPI in other anatomical locations. Our significant efforts to reduce HAPI, learn, and innovate have led us to examine the current impact of the learning health system model for this specific problem of interest.

## 2 METHODS

### 2.1 Setting

The setting for this project is an urban academic medical center located in the southeastern United States. The academic health center is the third largest public hospital in the United States. The medical center has 1207 beds, an average of 55 000 admissions per year, and 6000 ambulatory visits per day. The patient care is provided by 1400 physicians, 3600 nurses, and 800 advanced practice providers. Since 2002, this medical center has received ANCC Magnet^26^ status five consecutive times (in 2002, 2006, 2011, 2015, and 2019).

### 2.2 Institutional Review Board

Exempt Institutional Review Board approval was obtained from the organization's IRB for conducting this study.

### 2.3 Study design

In this descriptive, observational study, we retrospectively examined longitudinal data from October 1, 2019 to March 31, 2022 using the variables of total number of all‐stage HAPI counts and average length of stay (ALOS).

The case mix index, a marker of the hospital's patient acuity, has increased or remained constant. Our observed to expected (O/E) mortality also remained consistent during the time period. Because of our significant improvement history within the organization, we included HAPI statistical process control data from April 2018 to March 31, 2022, to align with our improvement efforts and provide contextual baseline data. Our findings will vary with the data from April 2018 to March 2022.

### 2.4 Data collection and analysis

An Excel^27^ spreadsheet was used by the team to collect organizationally reported counts of all stage HAPI and ALOS. HAPI data are collected from our electronic health record and stored in our HAPI analytic database, a business intelligence platform. Data for ALOS were obtained from the organization's financial decision support system, another business intelligence tool. The data were collected and reported monthly, using hospital level aggregates. We also analyzed patient characteristics, observed to expected (O/E) mortality ratios, as well as total patient days, and the case‐mix index to determine whether these factors varied over the study period.

We used the AHRQ cost estimates of $14 506 per event for HAPI.^28^ For this analysis, we used the fiscal year, October‐September (2020‐2021), and created an average number of events for each fiscal year, which was multiplied by the AHRQ estimates for each event. Total HAPI costs per fiscal year were then annualized for 12 months.

To analyze these data, the study team used the functionality within the Excel spreadsheet to create a summary, descriptive statistics, and graphs.^29^ We created statistical process control charts using an Excel add‐in, QI Macros.^30^ We also used SPSS^31^ to analyze the correlation between HAPI counts and ALOS.

## 3 RESULTS

**FIGURE 2:**
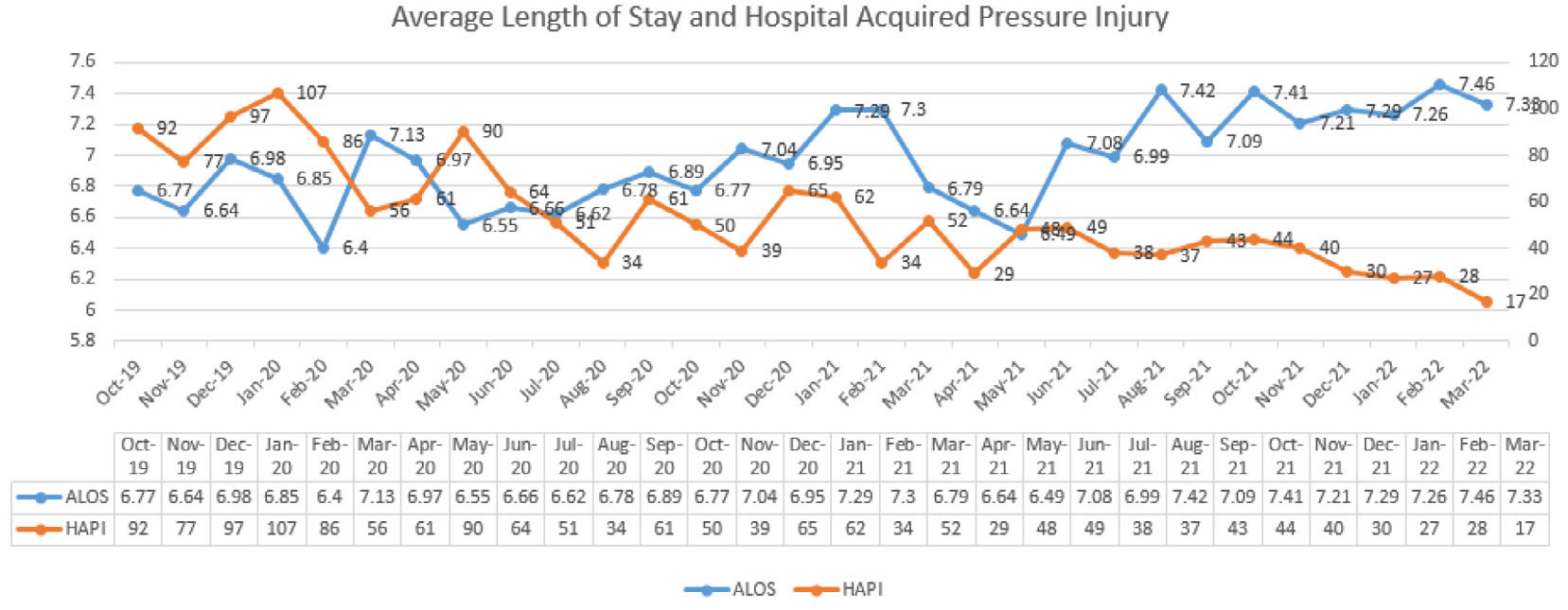
Average length of stay and HAPI incidence, 2019‐2022

Total patient days and ALOS remained consistent or increased since 2018, ranging from 392 550 (2018) to 431 812 (2021), with an average of 405 439, excluding the partial year of 2022 (292642), reflecting a relatively consistent patient volume. Changes in hospital volume are noted during the early months of the pandemic in 2020. From October 1, 2019 to March 31, 2022, the ALOS ranged from 6.40 to 7.46, an overall average of 6.97 for the time period. HAPI counts ranged from 17‐107, overall average of 53.6 for the same time period. HAPI per 1000 patient days for FY 20 (October‐September) and FY 21 decreased from 2.30 to 1.30 (Figure 2).

**FIGURE 3:**
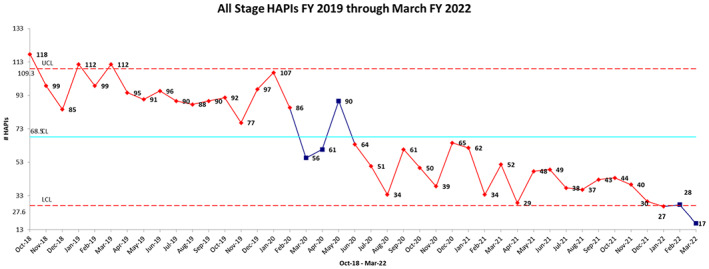
SPC for all stage HAPIs, 2019 to 2022

Our organization committed to a large‐scale improvement effort for HAPI beginning in 2016. The iterative improvements previously described created confirmed and valid data for all stages of HAPI beginning in 2018.^20^, ^21^ SPC showed special cause variation in 2019, 2020, and 2021 (Figure 3).

A correlation analysis was used to analyze the relationship between HAPI and ALOS (Table 2). A statistically significant, strong negative correlation, *r* = −.524, *P* = .003, was found.

**TABLE 2:** Correlation analysis for ALOS and HAPI

Correlations			
		ALOS	HAPI
ALOS	Pearson correlation	1	−0.524**
	Sig. (2‐tailed)		0.003
	N	30	30
HAPI	Pearson correlation	−0.524**	1
	Sig. (2‐tailed)	.003	
	N	30	30

** Correlation is significant at the 0.01 level (2‐tailed).

The annualized event AHRQ cost estimates for HAPI decreased from $12, 707, 256 to $7, 920, 276. A decrease of $4,786, 980 from FY 20 to FY 21.

## 4 DISCUSSION

### 4.1 Sustained improvement

The LHC efforts directed toward HAPI reduction have led to sustained improvements spanning multiple years. Longitudinal analysis using SPC shows special cause variation over the course of the last 3 years, indicating a continuous decline in HAPI counts. This reduction is particularly notable, given the worsening of other quality outcomes nationally during this same time period. For example, hospital acquired infection ratios were significantly higher in 2021 than 2019, with infection incidence worsening ranging from 39% to 149% compared to the pre‐pandemic period in 2019.^32^

Through our 6‐year improvement effort to mitigate HAPI, we utilized rapid cycle quality improvement, standardized processes and workflow, technology, leadership, and evidence‐based care to decrease the total all‐stage HAPI counts from baseline (March 2018, *n* = 164) to our lowest number (March 2022, *n* = 17), a 90% reduction, with a sustained reduction of greater than 30% per calendar year, despite an increase in ALOS over this time period. We believe that increases in ALOS, specifically for 2020‐2021 were likely attributable to the shift in care delivery to higher acuity COVID patients requiring longer hospitalizations associated with a lack of early effective treatments and limited prevention (vaccines), eventually evolving into emerging and experimental therapeutics that increased survivability but required hospitalizations, particularly in older and more chronically comorbid patients. In 2022, our numbers are currently trending below the lower sigma limit, denoting an early pattern of continued improvement. Our HAPI rate per 1000 patient days decreased by 43% from FY 20 to FY 21.

Conventional wisdom would question the statistically significant and negative correlation between ALOS and HAPI. Research has shown that patients with longer lengths of stay, or more time in a hospital setting, are more at‐risk for the development of HAPIs. However, this result creates what may be the strongest case for LHS‐guided quality efforts. Due to the significant continuous improvement and reduction in HAPI, it appears we have reversed the correlation. The reversed correlation reflects that our standardized processes and hard‐wired system of improvement has resulted in a consistent decrease in HAPI despite increases in our ALOS regardless of time spent in the hospital.

#### 4.1.1 The LHS differentiator

The sustained results noted in this study highlight an important differentiator between the LHS model and a more siloed or ad‐hoc process improvement approach. Key features of the LHS model include a culture of learning and a leadership commitment to continuous improvement. These features support the investment of resources with a longer time horizon than the current or upcoming fiscal year. The early investment in a 16‐member clinical WOCT team to address HAPI represented a large vote of confidence in the possibilities of the LHS model. While this investment has resulted in an estimated $4 million/year cost reduction, the results were not immediate. Additionally, the development of the HAPI analytical system was a long‐term investment that has reaped continued benefits and has supported the sustained improvement in outcomes noted across the study period. These investments in resources are unlikely to be supported without the leadership commitment to an ongoing and long‐term organizational interest in continued learning which is the hallmark of the LHS.

### 4.2 The value equation

The value equation in health care improves when an organization invests in improving quality, while either sustaining or reducing costs.^33^ Quality includes the clinical outcomes and the financial impact of improvement. Our organizational learnings related to value have been grounded in internal analysis of HAPI costs. Using ROM and SOI adjusted data, we found that our organizational costs associated with the care and management of HAPI are lower than literature estimates (Polancich et al., 2020),^22^ likely due to our prevention strategies and early management by our WOCT. In addition, there are costs associated with the reduction in reimbursement and the cost associated with care delivery specifically, such as additional intervention, therapeutics, and supplies.

For purposes of discussion, we estimated the cost of HAPI in alignment with the AHRQ literature estimates. Using the AHRQ approach, our significant and sustained reduction in HAPI is estimated to result in millions of dollars in savings annually. Although our structural resources have increased to expand our WOCT, if the AHRQ estimates for HAPI are accurate, the greater than $4 million cost avoidance from FY 20 to FY 21 with HAPI reduction would offset the cost of additional WOC Nurses, or at the very least create a budget neutral financial outcome.

Interprofessional collaboration is necessary for achieving sustainable improvements. While the human resource expertise of the WOC Nurses created the workflow and changed processes, adjusted the WOC staff‐to‐bed ratios, and built the interprofessional team to lead the charge; the proficiency of the database experts and improvement scientists who knew how to create sustainable change in a LHC was necessary. However, ultimately, to create a learning system, providers and stakeholders, specifically nurses, are essential to integrating standardized workflow and evidence‐based processes for HAPI reduction into practice.

### 4.3 Implications for practice

These results demonstrate the benefits of a holistic approach to quality improvement offered by the LHS model. The LHS model goes beyond a problem‐based approach to process improvement. Rather than targeting a specific problem to solve, the LHS system creates structures that yield sustained process improvement benefits over a prolonged time period. The early investment and development of these structures demonstrated their added benefit of positioning the organization for continued improvement during a period of national decline in overall safety and quality during the pandemic.^32^ These improvements have also resulted in cost savings during a period of financial strain noted in health systems nationwide.^34^ These findings highlight the benefit of a commitment to a learning infrastructure in bolstering organizational resilience and sustained performance when crisis hits. Leaders must advocate for and invest in structures that support continued organizational learning rather than emphasizing individual strategies for solving specific problems.

## 5 CONCLUSIONS

Becoming a learning health system is an iterative process, developing over time with strong leadership, effective use of data, and a culture and workforce committed to continuous learning and improvement. Developing a LHS culture and stance is imperative in our current health care landscape to effectively achieve safe, high‐quality care and outcomes. Despite the commitment that is required to evolve into a LHS, leaders effectively completing the journey may ultimately achieve value, defined as the highest quality of care delivery provided at the lowest cost, and an optimal patient experience. Our experience with HAPI reduction is evidence of an LHS transformational outcome.

## CONFLICT OF INTEREST

The authors report that they have no conflicts of interest.
